# Estimating genetic effect sizes under joint disease-endophenotype models in presence of gene-environment interactions

**DOI:** 10.3389/fgene.2015.00248

**Published:** 2015-07-28

**Authors:** Alexandre Bureau, Jordie Croteau, Christian Couture, Marie-Claude Vohl, Claude Bouchard, Louis Pérusse

**Affiliations:** ^1^Laboratoire de Biostatistique et Psychiatrie Génétique, Centre de Recherche de l'Institut Universitaire en Santé Mentale de QuébecQuébec, QC, Canada; ^2^Département de Médecine Sociale et Préventive, Université LavalQuébec, QC, Canada; ^3^Département de Kinésiologie, Université LavalQuébec, QC, Canada; ^4^Institut sur la Nutrition et les Aliments Fonctionnels, Université LavalQuébec, QC, Canada; ^5^École de Nutrition, Université LavalQuébec, QC, Canada; ^6^Human Genomics Laboratory, Pennington Biomedical Research CenterBaton Rouge, LA, USA

**Keywords:** abdominal obesity, endophenotype, familial association studies, generalized estimating equations, metabolic syndrome, physical activity, polytomous logistic model, transition model

## Abstract

Effects of genetic variants on the risk of complex diseases estimated from association studies are typically small. Nonetheless, variants may have important effects in presence of specific levels of environmental exposures, and when a trait related to the disease (endophenotype) is either normal or impaired. We propose polytomous and transition models to represent the relationship between disease, endophenotype, genotype and environmental exposure in family studies. Model coefficients were estimated using generalized estimating equations and were used to derive gene-environment interaction effects and genotype effects at specific levels of exposure. In a simulation study, estimates of the effect of a genetic variant were substantially higher when both an endophenotype and an environmental exposure modifying the variant effect were taken into account, particularly under transition models, compared to the alternative of ignoring the endophenotype. Illustration of the proposed modeling with the metabolic syndrome, abdominal obesity, physical activity and polymorphisms in the *NOX3* gene in the Quebec Family Study revealed that the positive association of the A allele of rs1375713 with the metabolic syndrome at high levels of physical activity was only detectable in subjects without abdominal obesity, illustrating the importance of taking into account the abdominal obesity endophenotype in this analysis.

## Introduction

Genome-wide association studies (GWAS) have been very successful at identifying genetic associations reproducible across multiple studies and meeting stringent criteria for statistical significance, but the effect sizes of the variants detected, measured by relative risks or odds ratios, are generally small (Altshuler et al., 2008). For genetic variants whose effect is modified by the environment, the effect estimated by GWAS is a marginal effect, i.e., an average of large effects in subjects with particular genotype-environment configurations and negligible effects in others. Estimating effects at specific levels of a key environmental exposure is crucial for lifestyle counseling taking into account genetic information. For instance, the knowledge that a genetic variant has an important protective effect against cardio-vascular disease only in the presence of a sufficient level of physical activity may be a further incentive to exercise regularly. Most studies of the modifying effects of environmental exposures have adopted crude stratifications of exposure to estimate genetic effects, but modeling genetic effects as a function of a quantitative exposure is likely to be more informative.

With complex traits, an affected/unaffected status constitutes a crude phenotypic dichotomy. Increasingly, multiple phenotypic measurements are used to decompose complex traits into components with a simpler etiology, which have been called endophenotypes (Gottesman and Gould, 2003; Szatmari et al., 2007). In some cases, syndromes are defined on the basis of impairments on multiple endophenotypes, such as the metabolic syndrome (MetS), which is defined from diabetes and cardiovascular risk factors (see Methods). The co-segregation of a disease and impairment in an endophenotype aggregating in families suggests genetic variants influencing both traits. This is one of the reasons why the present study takes advantage of familial association studies.

Endophenotypes are often used as univariate or multivariate phenotypes in genetic association studies, in place of the disease diagnosis. Since the ultimate goal is usually to estimate the effect of genetic variants on the risk of disease, it is of interest to jointly analyze the disease with one of its endophenotypes, and thus hopefully distinguish genetically distinct sub-types of an heterogeneous disease. In that case, a genetic variant may have a large effect on disease risk only when an endophenotype impairment is present or absent, in addition to require some level of an environmental exposure. We have previously developed within-family score tests under polytomous models, which can be used to jointly analyze the association between polytomous phenotypes defined from disease status and a dichotomous endophenotype, and two genetic polymorphisms in family samples (Bureau et al., 2014). While these tests designed to evaluate gene-gene interactions are applicable to a gene-environment setting with a dichotomous exposure, they do not provide effect size estimates.

In the present paper, we propose statistical modeling approaches to estimate genetic effects on the risk of disease taking into account an environmental exposure and a dichotomous endophenotype. Our goal is to compare genetic effect estimates and the power to detect them when jointly modeling disease and endophenotype as a function of exposure vs. ignoring the endophenotype in the analysis. This comparison is performed on genetic and environmental data simulated across a number of scenarios focused on MetS, abdominal obesity and physical activity in the Quebec Family Study (QFS), as well as actual genotype data from single nucleotide polymorphisms (SNPs) detected by GWAS of metabolic traits and phenotypic and physical activity data from the QFS.

## Methods

### Joint disease-endophenotype models

We considered two types of disease-endophenotype models: transition models and polytomous models.

#### Transition models

The endophenotype *Y*_1_ is supposed to precede the disease endpoint *Y*_2_. The probability of an impairment on the endophenotype *Y*_1_ = 1 is a function of predictor variables: genotype coded as allele count *X*, environmental exposure *E* and covariates *Z*. The probability of disease *Y*_2_ = 1 is a function of the endophenotype *Y*_1_ and predictor variables. We consider a logistic model for these two probabilities for comparability with the polytomous model, but note that other types of binomial models could be used, such as a log-binomial model. With a single covariate Z, the transition models can be written:

(1) $$\begin{array}{rcl} log\left(\frac{P\left[Y_{1}=1|E,X,Z\right]}{P\left[Y_{1}=0|E,X,Z\right]}\right) & = & \alpha_{0}+\alpha_{1}E+\alpha_{2}X\\ +\alpha_{3}EX+\alpha_{4}Z \end{array}$$

(2) $$\begin{array}{rcl} log\left(\frac{P\left[Y_{2}=1|Y_{1},E,X,Z\right]}{P\left[Y_{2}=0|Y_{1},E,X,Z\right]}\right) & = & \gamma_{0}+\gamma_{1}E+\gamma_{2}X+\gamma_{3}EX\\ +\gamma_{4}Y_{1}+\gamma_{5}Y_{1}E+\gamma_{6}Y_{1}X\\ +\gamma_{7}Y_{1}EX+\gamma_{8}Z \end{array}$$

The model for *Y*_2_ in Equation (2) is of interest in the context of examining the risk of disease. For the stratum without endophenotype impairment (*Y*_1_ = 0), the gene-environment interaction effect is γ_3_ and the genotype effect at exposure level *E* = *e* is γ_2_ + γ_3_e. Likewise, for the stratum with endophenotype impairment (*Y*_1_ = 1), the gene-environment interaction effect is γ_3_ + γ_7_ and the genotype effect at exposure level *E* = *e* is γ_2_ + γ_6_ + (γ_3_ + γ_7_)e. All these effects are interpretable as the log of odds ratios (ORs).

#### Polytomous models

In this type of model, the odds of each combination of *Y*_1_ and *Y*_2_ against a reference category (taken to be *Y*_1_ = 0, *Y*_2_ = 0) are modeled separately as functions of predictor variables with each their own coefficients. Taking the log gives the following logistic functions:

(3) $$\begin{array}{rcl} log\left(\frac{P\left[{Y_{1}=1,Y}_{2}=0|E,X,Z\right]}{P\left[Y_{1}=0{,Y}_{2}=0|E,X,Z\right]}\right) & = & \beta_{10}+\beta_{11}E+\beta_{12}X\\ +\beta_{13}EX+\beta_{14}Z \end{array}$$

(4) $$\begin{array}{rcl} log\left(\frac{P\left[{Y_{1}=0,Y}_{2}=1|E,X,Z\right]}{P\left[Y_{1}=0{,Y}_{2}=0|E,X,Z\right]}\right) & = & \beta_{20}+\beta_{21}E+\beta_{22}X\\ +\beta_{23}EX+\beta_{24}Z \end{array}$$

(5) $$\begin{array}{rcl} log\left(\frac{P\left[{Y_{1}=1,Y}_{2}=1|E,X,Z\right]}{P\left[Y_{1}=0{,Y}_{2}=0|E,X,Z\right]}\right) & = & \beta_{30}+\beta_{31}E+\beta_{32}X\\ +\beta_{33}EX+\beta_{34}Z \end{array}$$

If one were interested only in genetic and environmental effects on disease with and without endophenotype impairment, one could exclude subjects with endophenotype impairment but without disease (*Y*_1_ = 1, *Y*_2_ = 0), and define a trichotomous model using only Equations (4) and (5). Gene-environment interaction effects and genotype effects are defined for each of these phenotype category comparisons in a similar fashion. For Equation (5) comparing affected subjects with endophenotype impairment to unaffected subjects without endophenotype impairment, the interaction effect is β_33_ and the genotype effect at exposure level *E* = *e* is β_32_ + β_33_*e*. Again, these effects are interpretable as log-ORs.

These disease-endophenotype models were compared to a standard disease-only logistic model ignoring the endophenotype:

(6) $$\begin{array}{l} log\left(\frac{P\left[Y_{2}=1|E,X,Z\right]}{P\left[Y_{2}=0|E,X,Z\right]}\right)=\eta_{0}+\eta_{1}E+\eta_{2}X+\eta_{3}EX+\eta_{4}Z \end{array}$$

### Estimation

We opted for a population level estimation approach, using generalized estimating equations (GEEs) and empirical estimates of the variance of coefficient estimates (Zeger et al., 1988) to deal with dependence between genotype, exposure and outcome among related subjects. For transition models, we tried two types of working correlation structures: independence and relationship specific, i.e., with a distinct correlation estimated for each of the following relationship pairs: spouses, parent-offspring, siblings, avuncular, grandparent-grandchild and first cousins. The estimation was performed using the function geese from the R package geepack. For polytomous models, only the independence correlation structure was used. The estimation was performed using the function nomLORgee from the R package multgee. The empirical estimator of the variance-covariance matrix of the coefficient estimates, robust to misspecification of the correlation structure, was used in all cases.

### Testing strategy

We performed various kinds of Wald tests of model coefficients to assess the power to detect genotype and gene-environment interaction effects, where the genotype is coded as an allele count (allelic effect). Under transition models, tests under the disease model of Equation (2) were performed separately in subject with (*Y*_1_ = 1) and without (*Y*_1_ = 0) an impairment of the endophenotype, and a Bonferroni correction for performing two tests of each kind was applied. Under polytomous models, tests were performed for each of the logistic functions in Equations (3)–(5), and a Bonferroni correction for performing three tests of each kind was applied. Under the standard disease-only logistic model (6), there is only one equation, so only one test of each kind was performed with no Bonferroni correction. The testing strategy also depended on the nature of the exposure variable as outlined below.

#### Dichotomous exposure

We performed both a test of the interaction effect itself (e.g., a test of γ_3_ + γ_7_ in Equation (2) for the stratum with endophenotype impairment *Y*_1_ = 1), and a conditional test of the genotype effect given the exposure, which is the joint 2° of freedom test of the genotype effect in unexposed subjects and the interaction effect (Kraft et al., 2007). In the stratum with endophenotype impairment *Y*_1_ = 1, for instance, this is the joint test of γ_2_ + γ_6_ and γ_3_ + γ_7_. Within-family score tests for two locus models (Bureau et al., 2014) are readily transposable to one locus and a dichotomous exposure and performed in addition to Wald tests.

#### Continuous exposure

With a continuous exposure, it is not possible to perform the conditional test of the genotype effect given the exposure: the main effect coefficients of the genotype represent the genotype effect at an exposure value of 0, which may not be the most relevant exposure level to observe the effect of gene-exposure interaction. We adapted the conditional test to the continuous case by testing association at two representative levels of the exposure and correcting the significance level for performing two tests. We opted to estimate and test the genotype effect at the first and third quartiles of exposure as low and high levels.

### Study of metabolic syndrome (MetS) and physical activity motivating the models

Since the conception of our simulation scenarios was guided by the application to a GxE analysis of the MetS and abdominal obesity in relation with physical activity level in the QFS, we briefly review the characteristics of this syndrome, physical activity and the QFS before describing our simulation scenarios.

#### The quebec family study

The QFS cohort includes a total of 951 subjects from 223 families recruited in the Quebec City area, of which 754 (336 men, 418 women) had genotypes and measurements available on at least four traits entering the definition of MetS and on the physical activity score. All phenotypes related to the MetS were measured in QFS subjects as explained in previous reports (Bossé et al., 2007; Plourde et al., 2013). Briefly, body weight, height and waist circumference were measured following standardized procedures (Lohman et al., 1988). Blood samples were obtained after a 12-h overnight fast and cholesterol and triglyceride concentrations were determined enzymatically, while HDL-cholesterol was determined after precipitation of low-density lipoproteins, as previously described (Pérusse et al., 1989a). Plasma glucose was assessed enzymatically and insulin levels were measured by radioimmunoassay, as previously described (Rice et al., 1996). Systolic and diastolic blood pressure measurements were obtained according to the recommendations of the American Heart Association, as described previously (Pérusse et al., 1989b). All procedures were approved by the institutional review board of the Medical Ethics Committee of Laval University and all subjects gave their informed written consent to participate in the study.

#### Metabolic syndrome (MetS)

The MetS is a cluster of risk factors that are associated with a five-fold increase in the risk of type-2 diabetes and a two-fold increased risk of cardiovascular disease (Lanktree et al., 2013; Kaur, 2014; O'Neill and O'Driscoll, 2015). We consider it as our disease endpoint although it is usually considered as a precursor to full blown metabolic diseases. According to the National Cholesterol Education Program - Adult Treatment Panel III (2002), at least three of the following risk factors need to be present to be classified as having MetS: abdominal obesity (waist circumference ≥ 102 cm for men and ≥ 88 cm for women), elevated blood pressure (≥130/85 mm Hg), moderate hypertriglyceridemia (plasma triglyceride levels ≥1.7 mmol/l), reduced high-density lipoprotein cholesterol (HDL-C) levels (< 1.0 mmol/l men and < 1.3 mmol/l for women) and fasting hyperglycemia (≥ 6.1 mmol/l). Growing evidence (Després and Lemieux, 2006; Kloting and Bluher, 2014; Mathieu et al., 2014; O'Neill and O'Driscoll, 2015) suggests that excess body fat (particularly abdominal, which is associated with a proinflammatory state and insulin resistance) is a key component of the syndrome. Obesity, particularly abdominal obesity, which is often assessed by measuring waist circumference, is one of the most prevalent manifestations of MetS. Some causes of MetS without abdominal obesity are likely to differ from those of MetS with abdominal obesity, hence the interest in including abdominal obesity (present or absent) as an endophenotype when modeling MetS. The prevalence of MetS and abdominal obesity is well known to increase with age (Ervin, 2009; Janssen et al., 2011; Riediger and Clara, 2011), a relationship we also observed in the QFS (see regression coefficient estimates in the Supplementary Material). In QFS, the prevalence of MetS based on the above definition reached similar levels in men (23.3%) and women (21.5%), which is in line with the 2009–2011 Canadian Health Measures Survey which indicated that 22% of Canadians aged 18–79 years have MetS (24% in men and 20% in women). Results from twin and family studies reviewed elsewhere (Teran-Garcia and Bouchard, 2007; Lanktree et al., 2013; Stancakova and Laakso, 2014; O'Neill and O'Driscoll, 2015) indicate that both MetS and its defining risk factors, including abdominal obesity, are characterized by heritable components.

#### Physical activity

Low physical activity level is associated with increased prevalence of obesity and MetS, and we will use physical activity level to illustrate how an environmental exposure can modify the association of genetic variants with MetS. For the purpose of this study, we use a physical activity score derived from a 3-day activity record, as described elsewhere (Bouchard et al., 1983; Pérusse et al., 1989c). Briefly, using the activity record, subjects were asked to report the dominant activity for each 15 min period during 24 h by using a list of activities. Activities were grouped into 9 different categories (scores 1–9) based on the energy expenditure associated with each activity, a score of 1 representing a very low energy expenditure (sleeping or resting in bed) and a score of 9 representing high-intensity level physical activity. In the present study we used the sum of categories 5–9, i.e., the sum, over the 3 days, of 15-min periods coded as 5–9, as an indicator of moderate to vigorous physical activity. We previously showed evidence of familial aggregation for physical activity level measured by this score (Simonen et al., 2002). Evidence of familial co-aggregation has also been reported between features of MetS and physical activity (Butte et al., 2006; Vattikuti et al., 2012). The logit of the prevalence of MetS and the logit of the prevalence of abdominal obesity exhibit an overall decreasing relationship with the physical activity score (Supplementary Figures 1, 2), so we modeled the score linearly on the logistic scale.

### Simulation scenarios

We conducted a simulation study with the goals of confirming that the parameter estimates proposed above and associated standard errors and Wald test statistics have the expected statistical behavior. Simulation scenarios were inspired from data on MetS and abdominal obesity available in the QFS and our previous work (Bureau et al., 2014). A detailed description of the simulation scenarios can be found in the Supplementary Material. The following characteristics were taken into account:

- The prevalence of abdominal obesity and the prevalence of MetS with and without abdominal obesity (Table 1).
- The association between these prevalences and age.
- For scenarios with a continuous exposure, the relationship of the physical activity score with age.
- The familial correlation for MetS, abdominal obesity and the environmental exposure.
- The correlation between MetS, abdominal obesity and the environmental exposure.

**Table 1:** **Prevalence of metabolic syndrome and its component risk factors per abdominal obesity status among genotyped subjects from the Quebec Family Study**.

	**Prevalence**		**Abdominal obesity**	
	**N**	**%**	**Affected 220 (29.2%)**	**Non-affected 534 (70.8%)**
**MetS**				
Affected	150	19.9	133 (60.5%)	17 (3.2%)
Non-affected	604	80.1	87	517
**ELEVATED BLOOD PRESSURE**^a^				
Affected	186	24.7	98 (44.5%)	88 (16.5%)
Non-affected	567	75.3	122	445
**HYPERTRIGLYCERIDEMIA**				
Affected	214	28.4	113 (51.4%)	101 (18.9%)
Non-affected	540	71.6	107	433
**REDUCED HDL-CHOLESTEROL**				
Affected	332	44.0	149 (67.7%)	183 (34.3%)
Non-affected	422	56.0	71	351
**HYPERGLYCEMIA**^b^				
Affected	55	7.4	40 (18.3%)	15 (2.9%)
Non-affected	686	92.6	179	507

a *Data available for 533 subjects among the 534 non-affected of Abdominal obesity*.

b *Data available for 219 subjects among the 220 affected of Abdominal obesity and 522 subjects among the 534 non-affected of Abdominal obesity*.

Data were simulated under three joint models of MetS, abdominal obesity and physical activity:

- Transition model 1: where the interaction between the environmental exposure and a genetic polymorphism occurs only in subjects with abdominal obesity (*Y*_1_ = 1). For the scenario with a dichotomous exposure, obese subjects with the exposure form the group susceptible to a genetic effect on the risk of MetS (susceptible group).
- Transition model 2: where the interaction between the environmental exposure and a genetic polymorphism occurs only in subjects without abdominal obesity (*Y*_1_ = 0). For the scenario with a dichotomous exposure, non-obese subjects with the exposure form the susceptible group.
- A polytomous model where the gene-environment interaction is such that the genotype influences the onset of MetS (*Y*_2_ = 1) only among subjects with abdominal obesity (*Y*_1_ = 1), and where this influence depends on the level of exposure. For the scenario with a dichotomous exposure, obese subjects with the exposure form the susceptible group.

For all three scenarios, the exposure could either be a dichotomous variable with overall prevalence of 50% (e.g., a score dichotomized at the median) or a quantitative variable with distribution similar to the QFS physical activity score. The polytomous model with a dichotomous exposure is the simulation model used by Bureau et al. (2014) where the exposure is positively associated with *Y*_1_ and *Y*_2_, contrary to what was specified in transition models.

The genotype, dichotomous exposure or physical activity score, abdominal obesity and MetS variables were simulated in samples with the same size, family structures and age distribution as the QFS. Each simulated dataset was analyzed with the transition model, the polytomous model and a dichotomous model of *Y*_2_ (MetS) ignoring *Y*_1_ (abdominal obesity). Each dataset was therefore analyzed with a correctly specified model and two misspecified models. All simulations were repeated 1000 times.

## Results

### Simulation results

Analysis of a genetic variant independent from the risk variant revealed that the Wald tests involving transition model terms had approximately correct Type I error rates, except the tests in subjects without endophenotype impairment for the continuous exposure, which were noticeably liberal (Supplementary Table 1). We also noticed slightly higher Type I errors on datasets simulated from the polytomous model than on datasets simulated from transition models.

#### Dichotomous exposure

Table 2 shows the results of the simulation with a dichotomous exposure for the independence working correlation structure. Results for the relationship specific correlation structure were similar (not shown). With correctly specified models (e.g., data simulation and analysis under a transition model), the mean estimated genotype effects in the susceptible group are similar to the mean interaction effects, as expected since the genotype effect and interaction effect were identical under the simulation models (there was no genotype effect in the unexposed subjects, see Supplementary Material). The mean SEs of the interaction effect estimates are however much larger than the mean SEs of the genotype effect estimates, since interaction effect estimates represent a difference of genotype effect estimates between the exposed and unexposed groups, which is more variable than the estimated effect of the genotype in each of these two groups. As a result, the precision to estimate and the power to detect the genotype effect is greater than the precision to estimate and the power to detect the interaction effect. The power difference occurs both with the Wald test and the within-family score test. The population-level Wald test had generally greater power than the within-family score test, except for detecting interaction effects under some of the data generation—analysis model combinations (e.g., with data generated under the polytomous model, the within-family score test was more powerful in analyses using the polytomous and disease only model). Confidence interval coverage of the genotype and interaction effects under correctly specified models were close to nominal level (Supplementary Table 2).

**Table 2:** **Simulation results with a dichotomous exposure**.

**Analysis model**	**Genotype effect**						**Interaction effect**					
	**Effect simulated**	**Mean estimate**	**Mean SE**	**Estimate empirical SD**	**Power ^a^^,^^b^ conditional Wald**	**Power within family score**	**Effect simulated**	**Mean estimate**	**Mean SE**	**Estimate empirical SD**	**Power^b^ Wald**	**Power within family score**
**DATA SIMULATED UNDER TRANSITION MODEL 1**^c^												
Transition^d^	0.693	0.589	0.242	0.248	0.466	–	0.693	0.597	0.423	0.426	0.20	–
Polytomous^e^	–	0.413	0.190	0.199	0.335	0.236	–	0.266	0.336	0.340	0.06	0.118
Disease-only^f^	–	0.365	0.166	0.171	0.501	0.303	–	0.309	0.270	0.275	0.22	0.192
**DATA SIMULATED UNDER TRANSITION MODEL 2**^c^												
Transition^g^	0.693	0.607	0.263	0.275	0.420	–	0.693	0.626	0.413	0.427	0.22	–
Polytomous^h^	–	0.592	0.257	0.266	0.375	0.123	–	0.611	0.409	0.420	0.18	0.063
Disease-only^f^	–	0.285	0.167	0.169	0.319	0.190	–	0.228	0.270	0.275	0.12	0.128
**DATA SIMULATED UNDER POLYTOMOUS MODEL**^c^												
Transition^d^	–	1.480	0.294	0.286	0.999	–	–	1.48	0.479	0.478	0.80	–
Polytomous^e^	1.39	0.989	0.195	0.199	0.994	0.943	1.39	0.994	0.334	0.343	0.73	0.895
Disease-only^f^	–	0.860	0.170	0.173	1.000	0.934	–	0.864	0.269	0.272	0.90	0.922

a *Joint 2 d.f. test of the effect of the genotype in unexposed subjects and of the genotype-environment interaction effect*.

b *Significance level set to α = 0.05/2 = 0.025 for transition model, α = 0.05/3 = 0.0167 for polytomous model and α = 0.05 for disease-only model*.

c *See Section Dichotomous Exposure of the Supplementary Material*.

d *The estimated genotype log-odds ratio is γ_2_ + γ_6_ + γ_3_ + γ_7_ and the estimated interaction log-odds ratio is γ_3_ + γ_7_ from Equation (2)*.

e *The estimated genotype log-odds ratio is β_32_ + β_33_ and the estimated interaction log-odds ratio is β_33_ from Equation (5)*.

f *The estimated genotype log-odds ratio is η_2_ + η_3_ and the estimated interaction log-odds ratio is η_3_ from Equation (6)*.

g *The estimated genotype log-odds ratio is γ_2_ + γ_3_ and the estimated interaction log-odds ratio is γ_3_ from Equation (2)*.

h *The estimated genotype log-odds ratio is β_22_ + β_23_ and the estimated interaction log-odds ratio is β_23_ from Equation (4)*.

We note that the coefficient estimates were attenuated compared to the coefficient values specified in the simulation scenarios, as expected given they are population-averaged estimates, while the specified values are subject-specific (see Fitzmaurice et al., 2011, chapter 14 on the distinction between population averaged and subject-specific logistic model coefficients).

We also observed that the mean estimated genotype and interaction effects in the standard disease-only model ignoring the endophenotype *Y*_1_ are smaller than the corresponding effects under joint modeling of *Y*_1_ and *Y*_2_. For instance, when the data are simulated from transition model 2, the mean estimated log-OR for the genotype effect in the exposed subjects is 0.285 under the standard disease model, compared to 0.607 under the correct transition model and 0.592 under the polytomous model (category *Y*_1_ = 0, *Y*_2_ = 1). The interaction effect under the standard disease model is reduced to a greater extent than the genotype effect (0.228 compared to 0.626 under the correct transition model and 0.611 under the polytomous model). The transition model gives higher estimated effects than the polytomous model, even when the true underlying model is a polytomous model. The larger effect sizes under the joint models led to greater power to detect the genetic effect under transition model 2 (0.42 for the transition model vs. 0.32 for the disease only model), but not under transition model 1 (0.47 vs. 0.50). The polytomous model, which was taken from a previous study (Bureau et al., 2014), does not allow us to distinguish the power of the different approaches to detect the genotype effect, since the power is close to 1. To detect the interaction effect, the disease-only and transition models achieve higher power than the polytomous model, even though the data were generated under the latter. Power would have been slightly higher if we had tested the genotype and interaction effects using only Equations (4) and (5) and dividing the α level by 2 instead of 3 (ignoring subjects with endophenotype impairment but without disease).

#### Continuous exposure

**Figure 1:**
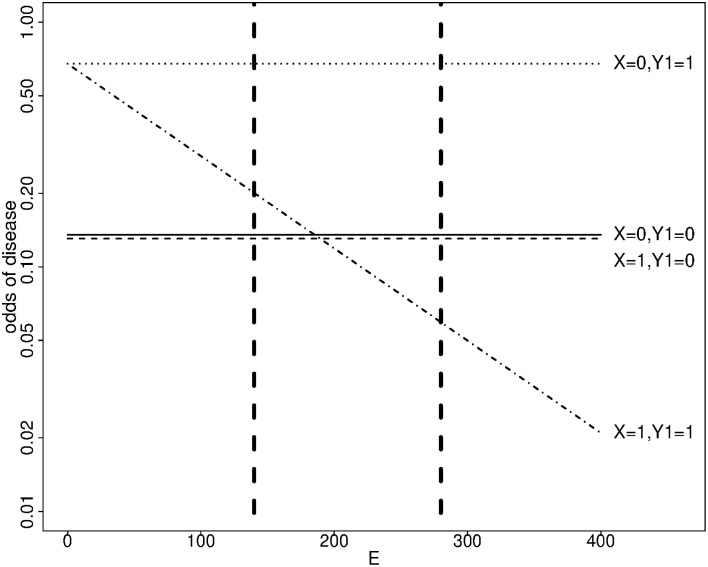
**Simulated odds of disease as a function of a genotype X and a continuous exposure E at age 40 under transition model 1**. X: number of minor alleles. *Y*_1_ = 1 if an endophenotype impairment is present, 0 otherwise. Dashed vertical lines show the first and third quartiles of E.

In our simulation scenarios, the variance of the simulated exposure corresponded to 0.6 times the variance of the physical activity score, and the first and third quartiles were 140 and 280 respectively. The effect of the minor allele was important at high exposure levels; it was protective under the transition models 1 and 2 (Figure 1), and deleterious under the polytomous model. We report the estimated genotype effects and the power at the third quartile along with the interaction effects for a change in exposure level equal to the exposure interquartile range (140 points) in Table 3. The genotype effects at the first quartile were low and power to detect them was small (not shown). The power advantage for the genotype effect test over the interaction test is even greater than in the dichotomous case, despite the Bonferroni correction for testing the genotype effects at two exposure levels. We note that the SE of the interaction coefficient under the transition and polytomous models underestimated the empirical SD on data simulated from transition models 1 and 2. However, no substantial inflation of the Type I error was detected (Supplementary Table 1). The mean SE was close to the empirical SD for the genotype effects for all simulation scenarios and for the interaction coefficients on data simulated from the polytomous model. As we observed with the dichotomous exposure, confidence interval coverage of the genotype and interaction effects under correctly specified models were close to nominal level (Supplementary Table 2).

**Table 3:** **Simulation results with a continuous exposure**.

**Analysis model**	**Genotype effect at 3rd quartile of exposure (280)**					**Interaction effect (for a 140 points change in exposure level)**				
	**Effect simulated**	**Mean estimate**	**Mean SE**	**Estimate empirical SD**	**Power^a^ Wald**	**Effect simulated**	**Mean estimate**	**Mean SE**	**Estimate empirical SD**	**Power^b^ Wald**
**DATA SIMULATED UNDER TRANSITION MODEL 1**^c^										
Transition^d^	−0.973	−0.894	0.240	0.251	0.901	−0.487	−0.446	0.268	0.316	0.220
Polytomous^e^	–	0.518	0.170	0.171	0.668	–	0.189	0.199	0.203	0.074
Disease-only^f^	–	−0.347	0.155	0.162	0.484	–	−0.201	0.187	0.199	0.203
**DATA SIMULATED UNDER TRANSITION MODEL 2**^c^										
Transition^g^	−0.973	−0.950	0.300	0.311	0.772	−0.487	−0.423	0.290	0.346	0.208
Polytomous^h^	–	−0.932	0.297	0.308	0.720	–	−0.411	0.332	0.347	0.159
Disease-only^f^	–	−0.203	0.154	0.154	0.175	–	−0.092	0.188	0.189	0.081
**DATA SIMULATED UNDER POLYTOMOUS MODEL**^c^										
Transition^d^	–	1.420	0.259	0.264	1.000	–	0.722	0.345	0.350	0.457
Polytomous^i^	1.433	0.950	0.178	0.179	0.997	0.647	0.443	0.240	0.244	0.282
Disease-only^f^	–	0.832	0.154	0.155	0.999	–	0.495	0.202	0.202	0.697

a *Significance level set to α = 0.05/4 = 0.0125 for transition model, α = 0.05/6 = 0.0083 for polytomous model and α = 0.05/2 = 0.025 for disease-only model*.

b *Significance level set to α = 0.05/2 = 0.025 for transition model, α = 0.05/3 = 0.0167 for polytomous model and α = 0.05 for disease-only model*.

c *See Section Continuous Exposure of the Supplementary Material. Note that the log-odds ratios in the Supplementary Material are reported for a 100 points change in exposure level*.

d *The estimated genotype log-odds ratio is γ_2_ + γ_6_ + 280(γ_3_ + γ_7_) and the estimated interaction effect is 140(γ_3_ + γ_7_) from Equation (2)*.

e *The estimated genotype log-odds ratio is β_12_ + 280β_13_ and the estimated interaction log-odds ratio is 140β_13_ from Equation (3)*.

f *The estimated genotype log-odds ratio is η_2_ + 280η_3_ and the estimated interaction log-odds ratio is 140η_3_ from Equation (6)*.

g *The estimated genotype log-odds ratio is γ_2_ + 280γ_3_ and the estimated interaction log-odds ratio is 140γ_3_ from Equation (2)*.

h *The estimated genotype log-odds ratio is β_22_ + 280β_23_ and the estimated interaction log-odds ratio is 140β_23_ from Equation (4)*.

i *The estimated genotype log-odds ratio is β_32_ + 280β_33_ and the estimated interaction log-odds ratio is 140β_33_ from Equation (5)*.

As in the dichotomous case, the mean estimated genotype and interaction effects in the standard disease-only model for *Y*_2_ignoring the endophenotype *Y*_1_ are smaller in absolute value than the corresponding effects under correct joint modeling of *Y*_1_ and *Y*_2_, and even under a misspecified joint model. For instance, when the data are simulated from transition model 2, the mean estimated log-OR for the genotype effect at an exposure level of 280 is −0.203 under the standard disease model compared to −0.950 under the correct transition model and −0.932 under the polytomous model (category *Y*_1_ = 0, *Y*_2_ = 1). The interaction effect is also reduced (−0.092 under the standard disease model, compared to −0.411 under the correct transition model and −0.423 under the polytomous model). Greater effect sizes led to greater power to detect the genotype effect with data generated from the transition models 1 and 2. For data generated from the polytomous model, the power to detect the genotype effect was near 1 for all methods applied, but we observed that the power to detect the interaction effect was greatest using the disease-only model. Again, defining the polytomous model using only Equations (4) and (5) would have slightly increased power under that model due to the larger α level.

When the data were simulated from transition model 1 and analyzed using the polytomous model, we note that the genotype and interaction effects on the endophenotype impairment among subjects without the disease (category *Y*_1_ = 1, *Y*_2_ = 0 vs. *Y*_1_ = 0, *Y*_2_ = 0) were detected with the greatest power, and these are the positive effects reported in Table 3. If a model without that category were used instead, the effects on the risk of both disease and endophenotype would be detected with the greatest power (power = 0.11 for the genotype effect and 0.07 for the interaction effect when applying the same α level as in Table 3).

### Illustration with physical activity, abdominal obesity and metabolic syndrome in the quebec family study

We illustrate the impact of jointly considering a disease (MetS) and an endophenotype (abdominal obesity) on the effect size and statistical significance of a genetic association at different levels of an environmental exposure using the QFS physical activity score as an example. Principal component analysis of the genotypes of 485,023 autosomal SNPs from the 610-Quad Illumina genotyping array on the QFS and HapMap samples using Eigensoft (Price et al., 2006) revealed no evidence of population stratification, the QFS subjects forming a tight cluster near the CEU HapMap sample. Concordingly, the variance inflation factor for the comparison of MetS affected and unaffected pedigree founders was estimated to be 1.000. We therefore decided not to perform any correction for population stratification.

**Figure 2:**
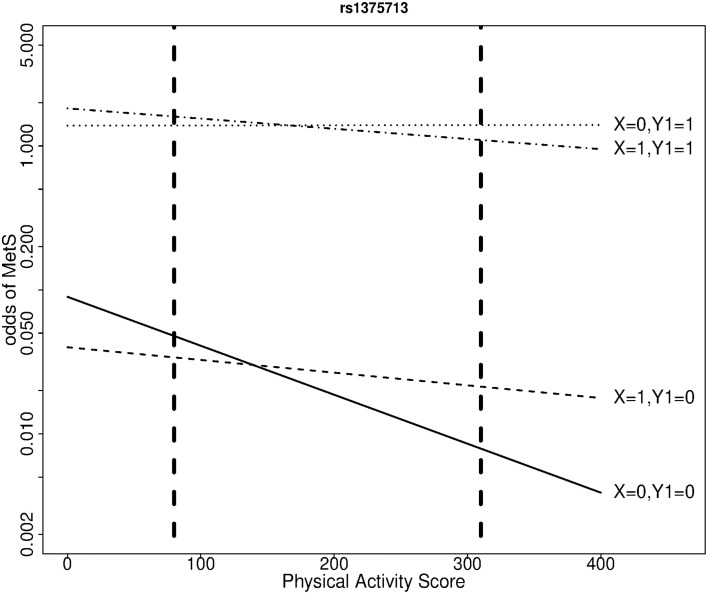
**Odds of metabolic syndrome as a function of moderate to strenuous physical activity score by rs1375713 genotype and abdominal obesity status, at age 40, in the QFS**. X: number of rs1375713 A alleles. *Y*_1_ = 1 if abdominal obesity, 0 otherwise. Dashed vertical lines show the first and third quartiles of the physical activity score.

We selected the gene *NOX3* for this illustration because the SNP rs9322557 was the most significantly associated to waist circumference in a genome-wide analysis (β = 0.27 cm/allele, *p* = 3.2 × 10^−7^) and this SNP and other showed evidence of association with various risk factors defining the MetS in the QFS. *NOX3* is part of the Nox family of NADPH oxidases, involved in generation of reactive-oxygen species (Leto et al., 2009) and in the development and progression of cardiovascular disease. The proposed joint modeling was applied to three *NOX3* SNPs: rs9322557, rs1375713, and rs12190809. We focus here on rs1375713 to illustrate the gain achieved with the proposed joint modeling. The SNP rs12190809 is strongly correlated to rs1375713 (*r*^2^ = 0.72) and gave similar results, while rs9322557 is weakly correlated to the other two (*r*^2^ = 0.07) and showed no evidence of interaction with physical activity level (not shown). Under a transition model, the interaction effect on MetS between the A allele of rs1375713 and physical activity level for a difference in physical activity score of 230 units (the interquartile range) was strong in subjects without abdominal obesity (OR [95% confidence interval (CI)] = 3.8 [1.4–10.0], *p* = 0.014) but absent among subjects with abdominal obesity (OR [95% CI] = 0.7 [0.4, 1.2], *p* = 0.4). Under a polytomous model, where the adjustment on age differs, the interaction effect on MetS in subjects without abdominal obesity was even stronger (OR [95% CI] = 4.1 [1.6, 10.4]). Without stratifying on abdominal obesity, no interaction effect with respect to MetS was detected (OR [95% CI] = 1.2 [0.7, 1.8], *p* = 0.5). Table 4 and Figure 2 help in interpreting this result. At low levels of moderate to strenuous physical activity, there is no difference in MetS prevalence as a function of the rs1375713 genotype, either in the subjects with or without abdominal obesity. Prevalence of MetS decreases with increasing levels of moderate to strenuous physical activity in subjects without abdominal obesity, but the decrease is accentuated in subjects not carrying the A allele (rs1375713 GG genotype), making this group susceptible to benefit most from moderate to strenuous physical activity. The rs1375713 A allele is hence attenuating the effect of physical activity on the prevalence of MetS in subjects without abdominal obesity, and can therefore be seen as a risk factor for MetS in active subjects without abdominal obesity (A allele OR [95% CI] = 2.7 [1.1, 6.5] at the 3rd quartile of the physical activity score). By contrast, no association of rs1375713 to MetS is detected at any level of the physical activity score when abdominal obesity is ignored. Finally, we note that when applying the same Bonferroni corrections as in the simulation study, the interaction test provided a significant result, but not the genotype effect test (Table 4), even though the genotype effect test is more powerful than the interaction test.

**Table 4:** **Association of the A allele of rs1375713 with metabolic syndrome depending on the level of moderate to strenuous physical activity and abdominal obesity status [normal or elevated waist circumference (WC)]**.

**Physical activity level**	**1st quartile (80 points)**			**3rd quartile (310 points)**		
	**OR**	**95% CI**	**P^a^**	**OR**	**95% CI**	**P^a^**
**TRANSITION MODEL (REFERENCE CATEGORY: NO MetS)**						
MetS (normal WC)	0.7	(0.4, 1.3)	1.0	2.7	(1.1, 6.5)	0.11
MetS (elevated WC)	1.2	(0.7, 2.0)	1.0	0.8	(0.5, 1.3)	1.0
**POLYTOMOUS MODEL (REFERENCE CATEGORY: NO MetS AND NORMAL WC)**						
MetS and normal WC	0.6	(0.3,1.3)	1.0	2.6	(1.1, 6.2)	0.16
MetS and elevated WC	0.9	(0.6, 1.3)	1.0	0.8	(0.5, 1.3)	1.0
**STANDARD MODEL (REFERENCE CATEGORY: NO MetS)**						
MetS	0.9	(0.6, 1.2)	0.89	1.0	(0.7, 1.5)	1.0

a *P-value after Bonferroni correction was applied as in Table 3*.

## Discussion

Our simulation results show that the estimate of the effect of a genetic variant on disease risk conditioned on an environmental exposure can be substantially higher when an endophenotype and an environmental exposure modifying the variant effect are taken into account in a gene-environment interaction context, compared to when the endophenotype is ignored. In an analysis of the interaction of *NOX3* SNPs and physical activity in relation to MetS and abdominal obesity, the association of the A allele of rs1375713 with MetS at high levels of physical activity was only detectable in subjects without abdominal obesity, illustrating the importance of taking into account the abdominal obesity endophenotype. In that subgroup, our results suggest that GG homozygotes would achieve the largest MetS prevalence reduction from physical activity. This gene was selected because of the association of one SNP with waist circumference in a genome-wide analysis, but it is another uncorrelated SNP whose association to MetS in subjects without abdominal obesity was modified by physical activity. This illustrate that gene-environment interaction tests are approximately independent of main effect tests (Dai et al., 2012).

We compared two joint modeling approaches for the disease and endophenotype: a transition model where the risk of disease depends on the endophenotype status, which can modify the genetic and environmental effects, and a polytomous model, where the combination of disease and endophenotype statuses defines four phenotypic categories. The transition model tended to produce log-OR estimates closer to the actual log-OR of the genetic variant in the subgroup where the variant had an effect, even when the true underlying model of the disease and endophenotype was a polytomous model, not a transition model. Considering in addition the simpler interpretation of the results from the transition model, we are led to favor that model for joint disease-endophenotype modeling in unascertained samples. We emphasize that the analysis under a transition model has two important advantages over a stratified analysis where the sample is split into subsamples with and without endophenotype impairment and a logistic regression of disease status is performed separately in the two subsamples using Equation (6). First, in a stratified analysis, distinct covariate coefficients η_4_ would be estimated in each stratum, leading to distinct covariate adjustments of the effects of interests, and difficulties in interpreting differences between strata. Second, when stratifying a familial sample, dependency between subjects from the same family in different strata would be ignored in the variance estimates, resulting in biased inference on differences between strata.

Environmental exposures such as physical activity levels are often measured quantitatively. Provided the relationships of a quantitative exposure with the odds of disease and endophenotype impairment are approximately linear, treating the exposure as a continuous variable in regression models is good modeling practice. We contend that plotting odds of disease as a function of exposure and estimating an effect of interest (here a genotype effect) at meaningful levels of the exposure under a model where the exposure is a continuous variable is preferable to the common practice in the epidemiological literature of dichotomizing exposure variables to estimate effects in exposure strata. In the present study, we chose to estimate genotype effects at the first and third quartiles of exposure, which revealed very different genotype effects.

Endophenotype are also often measured quantitatively. If the relationship between a quantitative endophenotype and the risk of disease is smooth, the quantitative endophenotype could be used in Equation (2) of the transition model, with Equation (1) being replaced by a linear model. Association with the disease could then be tested at meaningful levels of the quantitative endophenotype, and this may provide greater power than dichotomizing a quantitative endophenotype. We did not investigate this option in this work because it does not apply to the relationship between waist circumference and MetS, which is discontinuous at the threshold for abdominal obesity. At that threshold, the risk of MetS jumps, since subjects above the threshold have one more risk factor and are therefore closer to meeting the definition of MetS.

We elected to estimate the transition and polytomous models in unascertained familial samples using GEEs. The effects estimated by GEEs under logistic models are interpretable as population-level effects (Fitzmaurice et al., 2011), which are of interest in a population health perspective. These effects are attenuated compared to the subject-specific effects specified in the logistic mixed models used to simulate the data. We tried a working correlation matrix with relationship-specific correlations, but noticed no gain in precision when estimating these correlations compared to setting them to 0 under the independence working correlation matrix. Since familial correlations are not the focus of the proposed modeling, we favor the working independence assumption, as in that case estimation reduces to fitting logistic and polytomous models as if the observations were independent. It is well known that the coefficients of such models (except the intercept) are estimable under outcome-dependent sampling such as case-control sampling or ascertainment of family samples based on phenotype. An alternative to GEEs consists in estimating logistic and polytomous mixed models by maximum likelihood or Bayesian a posteriori distribution using a variety of numerical or Monte Carlo approximations to the likelihood function (Hartzel et al., 2001; Fitzmaurice et al., 2011). Specification of a familial correlation structure based on the kinship matrix for the random effects (derived from a polygenic background model for the trait liabilities) creates numerical problems, which are not fully solved (Wang et al., 2015). Estimation of polytomous models with random effects correlated between logistic functions is already difficult without kinship structure for the familial correlation (Hartzel et al., 2001). Taking ascertainment into account in the likelihood adds further numerical problems (Papachristou et al., 2011). These limitations led us to avoid logistic and polytomous mixed models. If one is interested in risk differences under a transition model, such effects can be estimated under a linear mixed model (Zhou and Stephens, 2012), but constraints may be required to keep predicted probabilities between 0 and 1. We focused instead on ORs, as these are more commonly used association measures with dichotomous traits. Another alternative is within-family conditional maximum likelihood estimation, with conditioning on children phenotypes and parental genotypes (Cordell et al., 2004; Dudbridge, 2008). We did not consider this alternative because the conditioning on phenotype prevents the specification of transition models, only polytomous models could have been estimated.

In addition to providing effect estimates, Wald hypothesis tests can be conducted using the empirical variance estimates. The power of these tests was compared to the power of a within-family score test derived from the conditional likelihood under polytomous models (Bureau et al., 2014). Wald tests were noticeably more powerful than within-family score tests when data were generated under transition models, but the Wald test power advantage was reduced when data were generated under polytomous models, and the within-family score test was even more powerful than the Wald test to detect interaction effects. For marginal effects, previous studies had observed lower power of within-family score tests than the population-level approach on case-control samples for prevalent traits (Laird and Lange, 2006).

Our results also revealed that a greater effect size does not always equate to a greater power. With data simulated under a polytomous model, the power to detect the genotype and interaction effects were similar or larger in analyses performed with a disease-only model ignoring the endophenotype than under transition or polytomous models. Estimating larger models comes with the price of lower precision of the estimates. We also noticed that Type I error of Wald tests can be inflated when modeling a continuous exposure with a subgroup of limited size, such as the disease cases in the stratum without endophenotype impairment in our simulations, whose prevalence was based on MetS cases without abdominal obesity in the QFS (Table 1).

The QFS has several advantages for a study of the interaction between genes and physical activity in relation to MetS, including a detailed assessment of physical activity levels, extensive metabolic traits measurements, and absence of population stratification confounding in the Quebec City population of French-Canadian ancestry. In spite of this, the size of the QFS is limited and estimates lack precision for stratum-specific effects involving third order interaction terms in the transition models, or four phenotypic categories in the polytomous models. The current study was also limited to three SNPs in the *NOX3* gene. Replication of the findings reported herein will be needed before they can be used to promote lifestyle modifications in the subgroup of subject with MetS without abdominal obesity.

In summary, to identify circumstances under which genetic variants are strongly associated to a disease, we recommend estimating distinct genotype effects in subjects with and without impairment on a key endophenotype, at representative levels of environmental exposures influencing the trait of interest, under a joint model of the disease and the endophenotype, preferably the transition model. This study suggests that such strategy can be successful when both the environmental exposure and the endophenotype modify the genetic effect. Implementation of such modeling requires extensive phenotyping and environmental exposure characterization, typically available only on moderate size samples. In such setting, the proposed strategy should be focused on the most promising candidate genes, to limit the multiple testing penalty. Consistent phenotypic and environmental measurements across studies are required to envision implementing this strategy on a genome-wide scale in very large samples.

### Conflict of interest statement

The authors declare that the research was conducted in the absence of any commercial or financial relationships that could be construed as a potential conflict of interest.
