# Supplementary material for: Estimating genetic effect sizes under joint disease-endophenotype models in presence of gene-environment interactions
Source: Front Genet. 2015 Jul 28;6:248. doi: 10.3389/fgene.2015.00248 (PMC4516976; doi:10.3389/fgene.2015.00248)
Supplement: Supplementary file 1 [file DataSheet1.PDF]

frontiers

Supplementary Material

Estimating genetic effect sizes under joint disease-endophenotype models in presence of gene-environment interactions

Alexandre Bureau[1,2]*, Jordie Croteau[1], Christian Couture[3], Marie-Claude Vohl[4,5], Claude Bouchard[6] and Louis Pérusse[3,4]

[1]Laboratoire de biostatistique et psychiatrie génétique, Centre de recherche de l'Institut universitaire en santé mentale de Québec, Québec, Québec, Canada
[2]Département de médecine sociale et préventive, Université Laval, Québec, Québec, Canada
[3]Département de kinésiologie, Université Laval, Québec, Québec, Canada
[4]Institut sur la nutrition et les aliments fonctionnels, Université Laval, Québec, Québec, Canada
[5]École de Nutrition, Université Laval, Québec, Québec , Canada.
[6]Human Genomics Laboratory, Pennington Biomedical Research Center, Baton Rouge, LA, USA.

*** Correspondence:** Alexandre Bureau, Social and preventive medicine, Laval University, Pavillon Ferdinand-Vandry, 1050, avenue de la Médecine, Local 2457, Quebec city, Quebec, G1V 0A6, Canada.
alexandre.bureau@msp.ulaval.ca

1. Description of simulation scenarios

1.1 Random effects to capture familial correlation

Familial correlation due to polygenic background and shared environment was simulated using correlated random effects. For the phenotypes Y_1 and Y_2 we opted for multivariate standard normal polygenic effects U_1 and U_2, with pairwise correlation among relatives proportional to their kinship, and no shared environment. For the environmental exposure E, we opted instead for a multivariate standard normal environmental effect U_3, with equal correlation among all pairs of relatives, the value of which depended on the nature of the exposure. The random effects U_1, U_2 and U_3 for Y_1, Y_2 and E within the same subject were correlated to reflect epidemiological evidence for metabolic syndrome (MetS), abdominal obesity and physical activity correlation with the following correlation matrix:

	U_1	U_2	U_3
U_1	1	0.7	0.5
U_2	0.7	1	0.5
U_3	0.5	0.5	1

The random effects for the members of each family were drawn from a multivariate normal distribution with mean vector 0, unit variance and a correlation matrix constructed as described above.

1.2 Genotype

We simulated a biallelic variant with minor allele frequency = 0.3 and defined X as the number of minor alleles, taking values 0, 1 or 2. To verify the Type I error of tests under the null hypothesis of no association to the tested genetic variant, but in presence of genetic linkage with the risk variant, we generated an additional biallelic variant with the same minor allele frequency but independent from the causal variant, i.e. in linkage equilibrium with it.

1.3 Dichotomous exposure

We simulated the dichotomous E from the logistic mixed model

$$log\left(\frac{P[E=1|age]}{P[E=0|age]}\right) = 0.05 + 0.03age + U_3$$

where *age* was centered at 40 years and the intercept was chosen to achieve an overall 50% prevalence of the exposure in the sample. The correlation of U_3 among relatives was 0.3.

For transition models, the endophenotype status Y_1 was simulated from the logistic mixed model

$$log\left(\frac{P[Y_1=1|E,X,Z]}{P[Y_1=0|E,X,Z]}\right) = -1.5 + 0.693E + 0.203X + 0.030age + U_1$$

where the age coefficient equals the estimate in an univariate logistic regression of abdominal obesity on age and the intercept was chosen to approximate the prevalence of abdominal obesity in the QFS. Then Y_2 was simulated under two different models:

1) $log\left(\frac{P[Y_2=1|Y_1,E,X,Z]}{P[Y_2=0|Y_1,E,X,Z]}\right) = -2.5 + 1.609Y_1 + 0.693EXY_1 + 0.042age + U_2$
2) $log\left(\frac{P[Y_2=1|Y_1,E,X,Z]}{P[Y_2=0|Y_1,E,X,Z]}\right) = -2.5 + 0.693EX + 1.609Y_1 - 0.693EXY_1 + 0.042age + U_2$
where the age coefficient equals the estimate in an univariate regression of the logit of MetS on age and the intercept was chosen to approximate the prevalence of MetS in the QFS.

The polytomous model is similar to the one used in Bureau et al.(2014). The effect of X corresponds to the effect of the genetic variable X_2 in Bureau et al. (2014), except that coefficients are divided by 2 because X is coded 0,1 and 2 while X_2 was previously coded 0, ½, 1. The effect of E corresponds to the effect of the genetic variable X_1 in Bureau et al. (2014), but its prevalence is higher. Model intercepts were modified to approximate the abdominal obesity and MetS joint distribution in the QFS. For the polytomous model, we also simulated sequentially Y_1 and Y_2 even though the model gave us a joint distribution for these two variables, because the random effects were simulated for each variable separately. The simulation model for Y_1 was obtained by marginalizing over Y_2:

$$log\left(\frac{P[Y_1=1|E,X,Z]}{P[Y_1=0|E,X,Z]}\right) = log\left(\frac{\exp(-2 + 0.693E - 0.347EX) + \exp(-2.5 + 1.386EX)}{1 + \exp(-2.5)}\right) + 0.03age + U_1$$

Then Y_2 was simulated conditional on Y_1 :

$$log\left(\frac{P[Y_2 = 1|Y_1, E, X, Z]}{P[Y_2 = 0|Y_1, E, X, Z]}\right) = -2.5 + 2.0Y_1 - 0.693EY_1 + 1.733EXY_1 + 0.042age + U_2$$

1.4 Continuous exposure

For the transition models, we simulated E from the linear model

$$E = 2.098 + 0.0042age + U_3$$

where *age* was centered at 40 years. These coefficients were chosen to approximate the age-dependent mean of the moderate to strenuous physical activity score in the QFS divided by 100. The correlation of 0.16 among relatives for U_3 was the value estimated for the physical activity score in the QFS (Simonen et al., 2002).

For transition models, the endophenotype status Y_1 was simulated from the model

$$log\left(\frac{P[Y_1 = 1|E, X, Z]}{P[Y_1 = 0|E, X, Z]}\right) = -0.800 - 0.166E + 0.203X + 0.033age + U_1$$

where the E and age coefficient equal the estimates in a multiple logistic regression of abdominal obesity on age and the physical activity score and the intercept was chosen to approximate the prevalence of abdominal obesity in the QFS. Then Y_2 was simulated under two different models:

1) $log\left(\frac{P[Y_2=1|Y_1,E,X,Z]}{P[Y_2=0|Y_1,E,X,Z]}\right) = -2 + 1.609Y_1 - 0.348Y_1EX + 0.043age + U_2$

2) $log\left(\frac{P[Y_2=1|Y_1,E,X,Z]}{P[Y_2=0|Y_1,E,X,Z]}\right) = -2 - 0.348EX + 1.609Y_1 + 0.348Y_1EX + 0.043age$

where the age coefficient equals the estimate in a multiple logistic regression of MetS on age and the physical activity score, the coefficient for Y_1EX in transition model 1 and the coefficient for *EX* in transition model 2 are four times the coefficient estimate for the physical activity score in the same logistic regression. In transition model 2, the coefficient for Y_1EX was the opposite of the coefficient for *EX.* The intercept was chosen to approximate the prevalence of MetS in the QFS.

The polytomous model with a continuous exposure was derived from the polytomous model with a dichotomous exposure, by dividing the terms involving the exposure *E* by 3, in order to achieve at an exposure score of 3 (300 rescaled to original physical activity score) the same effect as in the exposed group with a dichotomous exposure. The simulation model for Y_1 was obtained by marginalizing over Y_2:

$$log\left(\frac{P[Y_1 = 1|E, X, Z]}{P[Y_1 = 0|E, X, Z]}\right) = log\left(\frac{\exp(-2 + 0.231E - 0.116EX) + \exp(-2.5 + 0.462EX)}{1 + \exp(-2.5)}\right) + 0.033age + U_1$$

Then Y_2 was simulated conditional on Y_1 :

genetic effect sizes under disease-endophenotype models with gene-environment interaction

$$log\left(\frac{P[Y_2 = 1|Y_1, E, X, Z]}{P[Y_2 = 0|Y_1, E, X, Z]}\right) = -2.5 + 2.0Y_1 - 0.231E + 0.231EY_1 + 0.578EX - 0.578EXY_1 + 0.043age + U_2$$

2. Supplementary Tables

Supplementary Table 1: Type I error estimates

	E dichotomous[a]				E continuous[b]			
	Genotype		Interaction		Genotype		Interaction	
	Y_1=1	Y_1=0	Y_1=1	Y_1=0	Y_1=1	Y_1=0	Y_1=1	Y_1=0
	Data simulated under transition model 1							
α = 1.25%	0.015	0.015	0.019	0.017	0.010	0.018	0.014	0.017
α = 2.5%	0.031	0.024	0.024	0.033	0.025	0.037	0.024	0.033
α = 5%	0.052	0.049	0.051	0.060	0.055	0.066	0.059	0.069
	Data simulated under transition model 2							
α = 1.25%	0.017	0.017	0.013	0.012	0.008	0.022	0.015	0.021
α = 2.5%	0.031	0.029	0.031	0.023	0.021	0.035	0.030	0.046
α = 5%	0.062	0.054	0.070	0.053	0.045	0.072	0.067	0.073
	Data simulated under polytomous model							
α = 1.25%	0.016	0.016	0.019	0.018	0.020	0.024	0.016	0.023
α = 2.5%	0.025	0.036	0.033	0.034	0.035	0.038	0.034	0.043
α = 5%	0.058	0.062	0.066	0.061	0.068	0.069	0.060	0.074

[a] Proportion of replicates where Wald statistics for the genotype log-odds ratio $\gamma_2 + \gamma_6 + \gamma_3 + \gamma_7$ (Y_1 = 1) and $\gamma_2 + \gamma_6$ (Y_1 = 0), and the interaction log-odds ratio $\gamma_3 + \gamma_7$ (Y_1 = 1) and γ_3 (Y_1 = 0) from Equation 2 rejected the null hypothesis at the specified Type I error levels in 1000 replicates of the analysis of a biallelic polymorphism tightly linked to but in linkage equilibrium with the risk polymorphism in the dichotomous exposure version of the specified model.

[b] Proportion of replicates where Wald statistics for the genotype log-odds ratio $\gamma_2 + \gamma_6 + 280(\gamma_3 + \gamma_7)$ (Y_1 = 1) and $\gamma_2 + 280\gamma_3$ (Y_1 = 0), and the interaction log-odds ratio 140 $(\gamma_3 + \gamma_7)$ (Y_1 = 1) and $140\gamma_3$ (Y_1 = 0) from Equation 2 rejected the null hypothesis at the specified Type I error levels in 1000 replicates of the analysis of a biallelic polymorphism tightly linked to but in linkage equilibrium with the risk polymorphism in the continuous exposure version of the specified model.

Supplementary Table 2: 95% confidence interval coverage of the mean estimates from Tables 2 and 3 for the analyses with correctly specified models.

	Genotype		Interaction	
	E dichotomous (E=1)	E continuous (E=280)	E dichotomous	E continuous
Transition model 1	0.952	0.941	0.959	0.934
Transition model 2	0.932	0.938	0.937	0.936
Polytomous model	0.943	0.945	0.950	0.948

3. Supplementary Figures

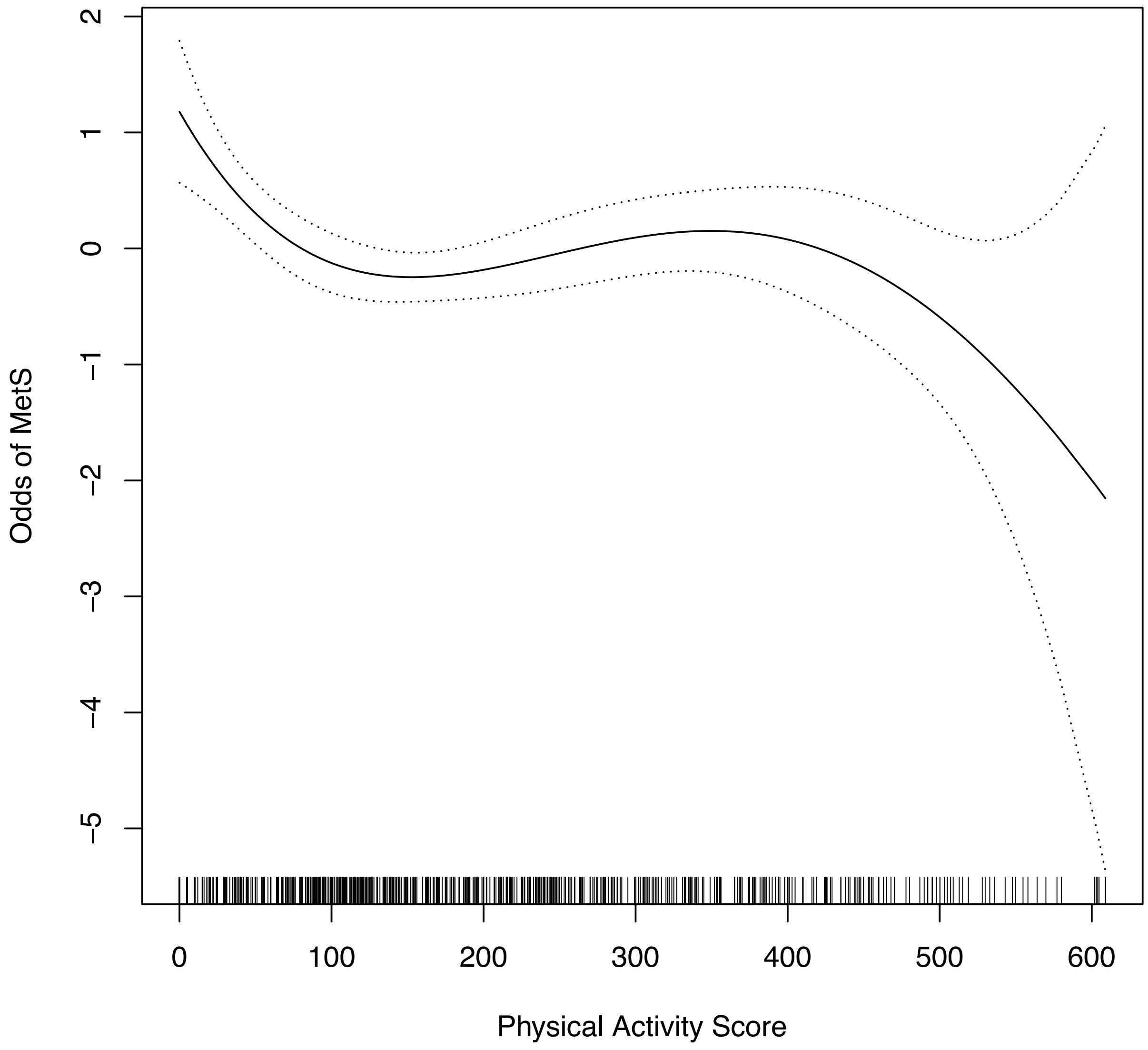

Supplementary Figure 1. Logit of the prevalence of MetS as a function of the physical activity score in the QFS using a polynomial fit of 4th order.

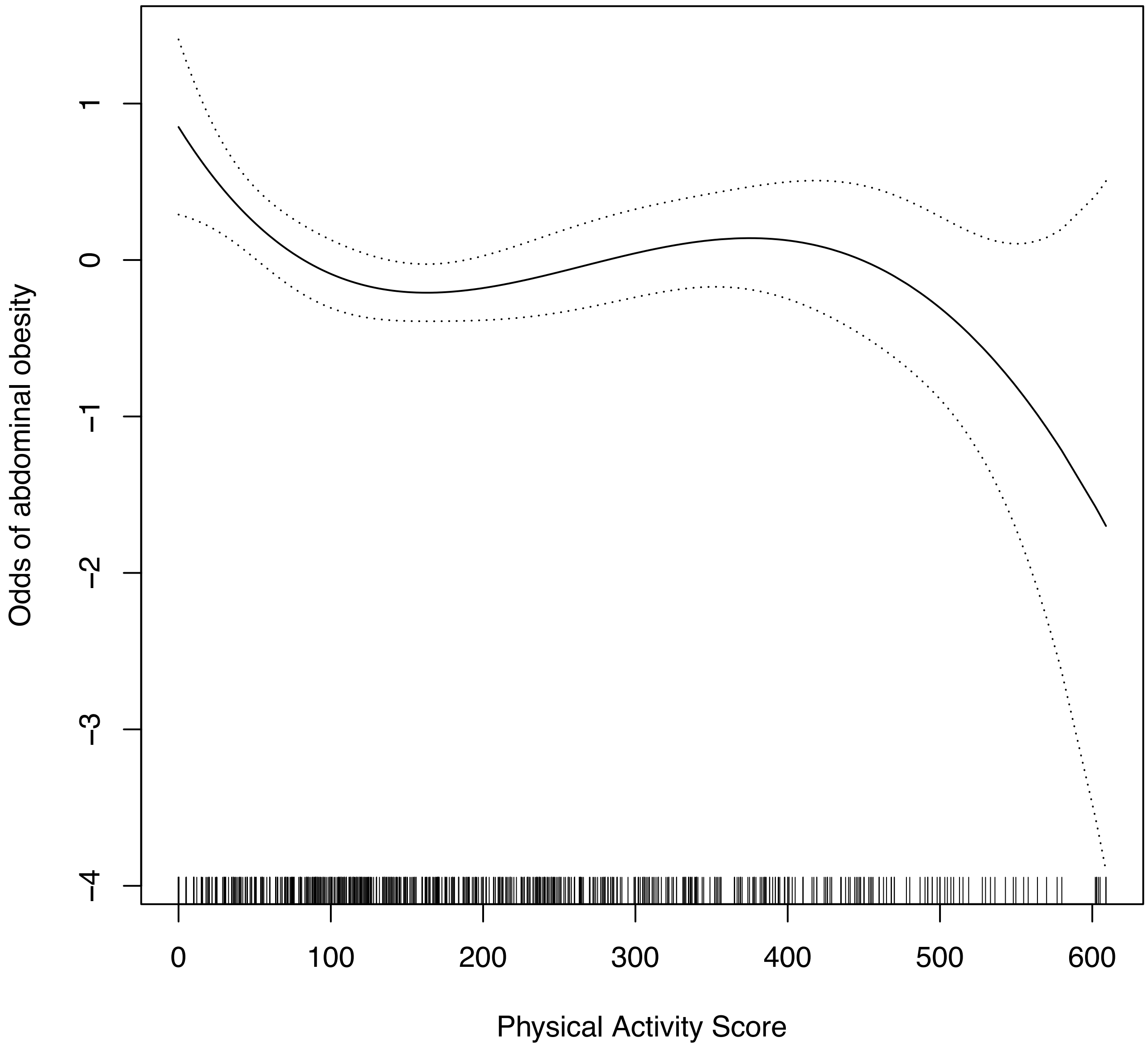

Supplementary Figure 2. Logit of the prevalence of abdominal obesity as a function of the physical activity score in the QFS using a polynomial fit of 4th order.

4. References

Bureau, A., Croteau, J., Chagnon, Y.C., Roy, M.A., and Maziade, M. (2014). Extension of the generalized disequilibrium test to polytomous phenotypes and two-locus models. *Front Genet* 5, 258. doi: 10.3389/fgene.2014.00258

Simonen, R.L., Perusse, L., Rankinen, T., Rice, T., Rao, D.C., and Bouchard, C. (2002). Familial aggregation of physical activity levels in the Quebec Family Study. *Med Sci Sports Exerc* 34, 1137-1142.
